# Assessment of Human Ambulatory Speed by Measuring Near-Body Air Flow

**DOI:** 10.3390/s100908705

**Published:** 2010-09-20

**Authors:** Alberto G. Bonomi, Stefano Salati

**Affiliations:** Care & Health Applications, Philips Research Laboratories, Eindhoven, The Netherlands; E-Mail: stef.salati@gmail.com

**Keywords:** walking speed, Pitot tube, wearable sensor, accelerometer

## Abstract

Accurate measurements of physical activity are important for the diagnosis of the exacerbation of chronic diseases. Accelerometers have been widely employed in clinical research for measuring activity intensity and investigating the association between physical activity and adverse health conditions. However, the ability of accelerometers in assessing physical activity intensity such as walking speed has been constrained by the inter-individual variability in sensor output and by the necessity of developing unobtrusive low-power monitoring systems. This paper will present a study aimed at investigating the accuracy of a wearable measuring system of near-body air flow to determine ambulatory speed in the field.

## 1. Introduction

Chronic adverse health conditions such as cardiovascular diseases, chronic obstructive pulmonary diseases (COPD) and many others lead to poor exercise tolerance and reduced levels of physical activity [1]. Patients with COPD spend less time walking in daily life than age-matched subjects and walk at a lower intensity [2]. Heart and vascular diseases are associated with reduced number of steps taken daily as compared to healthier conditions [1]. A marked decrease in physical activity usually indicates acute health deteriorations thereby increasing hospitalization and mortality risk [3,4]. Thus, physical performance is an essential prognostic parameter in many patient populations.

Walking is considered as one of the most frequent daily activity and the number of steps taken daily is a strong indicator of health status [1,5]. A tool often used in clinical practice to assess functional capacity in heart failure and COPD patients is the 6-minute walking test, which consists in a walking trial along a straight and levelled path at self selected intensity [6]. The outcome of such test is the 6-minute walking distance which reflects the patient ability to perform routine daily activities and it evaluates the global and integrated responses of all the systems involved during physical activity, including the pulmonary and cardiovascular systems [7,8]. This suggests that monitoring and detecting trends in walking speed in daily life might improve prevention and diagnosis for exacerbation of chronic diseases.

The development of portable sensor technology for measuring physical activity in free-living individuals has enabled the assessment of physical performance during daily activities outside the laboratory. Activity monitors based on acceleration sensors, also called accelerometers, have been developed and frequently tested for detecting type, duration and intensity of physical activity [9–14]. Traditionally accelerometer output was quantified in activity counts, which can be calculated as the sum of the rectified acceleration signal over epochs of one minute [15]. Activity counts have been used to characterize physical activity intensity according to cut-off threshold values by distinguishing periods of low-, moderate- or high-intensity activities. In addition, Levine *et al* [12] reported a within-individual log-linear relationship between activity counts and walking speed (r^2^ > 0.99). However, because of between-subjects differences in accelerometer’s output, generalized prediction models of walking speed were less accurate. The amount of activity counts recorded at specific walking speeds had a subjective variability of 10% [12]. More recently, activity recognition techniques have been developed to identify walking events and the engagement in different types of activities using the signal recorded with an accelerometer [9,16–19]. Temporal and spectral features of the acceleration signal have been used to estimate walking speed by developing multiple-linear regression equations [9]. Moreover, accelerometers-based methods have been presented to estimate walking speed by detecting gait events using pattern recognition techniques. Yet, subjects’ characteristics were necessary to improve the speed estimation accuracy [20]. Nowadays, the most accurate estimation of ambulatory speed in the field is provided by systems constituted by accelerometers and global positioning systems (GPS). These monitors are able to determine ambulatory speed during outdoors activities with accuracy of 0.08 km/h, independently from inter-individual differences in gait pattern [21,22]. However, GPS are often considered too expensive to be implemented in simple activity monitor systems, they are extremely power consuming in high frequency applications, and cannot be used indoors [23].

In this paper is presented a study aimed at testing the accuracy of an activity monitor able to measure the body acceleration and the near-body air flow during walking for estimating ambulatory speed in laboratory and field conditions. The hypothesis was that measurements of near-body air flow could improve the assessment of walking speed by reducing the inter-individual variability in sensor output during walking.

## 2. Methods

### 2.1. Instrumentation

**Figure 1.**
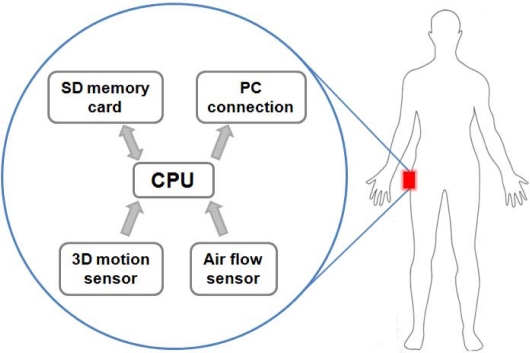
Scheme of the activity monitor used to measure acceleration and near-body air flow.

A wearable physical activity monitor was designed to measure Near-Body Air Flow (NBAF) and the body acceleration during movement. In addition to the sensing elements, the instrument included an ultra-low-power microcontroller (MSP430, Texas Instruments), a memory card for data storage (SD or MMC), a serial communication interface to transfer the acquired data to personal computer, and a battery unit allowing an operational lifetime of several days (3.2mW of measured power consumption) (Figure 1). The sensing part of the device was composed by a NBAF measurement system and a tri-axial accelerometer.

**Figure 2.**
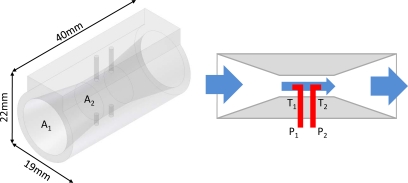
Design of the air duct and representation of the principle of the Pitot tubes of the near-body air flow system.

The NBAF system was developed to measure the speed of the air flowing near the body during ambulation and was made up by two parts: a modified Pitot-static tube inside an air duct and a MEMS differential pressure sensor (MB-LPS1-01-100B5N, Microbridge Technologies, Canada, Montreal). The air duct was designed with tapered mouths for enhancing the speed of the flowing air and for increasing the sensitivity of measure (Figure 2). It was designed using Autodesk Inventor 2008 (Autodesk Inc., Barendrecht, The Netherlands) and realized in ABS by a 3D printer (Shapeways, Eindhoven, The Netherlands). Inside the duct, the Pitot consisted of a tube pointing into the direction of the flowing air (T_1_) and a second one pointing in the opposite direction (T_2_). The tube pointing in the direction of the flowing air was used to measure the stagnation pressure, resulting from the sum of the atmospheric pressure and the dynamic pressure (related to air speed). The second tube was used as a reference, to measure the static pressure due to atmospheric factors (Figure 2). The higher the air speed inside the duct the higher the differential pressure between the tubes of the Pitot system. The relationship between the speed of the air flowing through the NBAF system (*v_NBAF_*) and the differential pressure can be described by:

(1) $$v_{\mathrm{NBAF}}\;=\;\frac{A_{2}}{A_{1}}\sqrt{\frac{2\Delta P}{\rho }}$$

where A_1_ and A_2_ are the areas of the outer and inner section of the tube, *ρ* the air density, and ΔP the differential pressure. Thus, the higher the walking speed the higher the speed of the air flowing inside the NBAF system, and thereby the bigger the measured ΔP.

**Figure 3.**
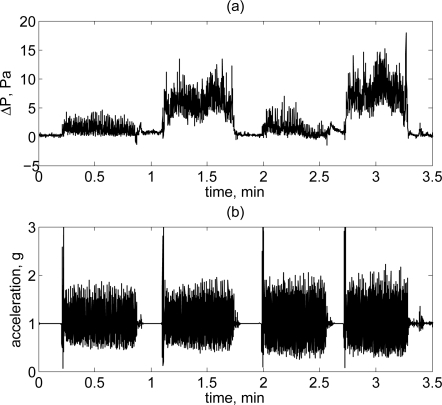
Representation of the readout signal of the activity monitor. **(a)** Differential pressure generated from air flowing through the Pitot tube during locomotion in outdoors conditions. **(b)** Acceleration measured during walking outdoors, the peak in the acceleration pattern preceding the walking signal results from a jumping-jack executed to identify the start of a new event.

The ΔP generated inside the duct was measured using a bidirectional pressure sensor, with full scale of ±250 Pa and a very high flow-impedance which allowed the minimization of the flow through the tubes of the Pitot system. The transducer was a MEMS-based thermo-anemometer on a monolithic silicon chip. The system had a very short response time, in the order of 2–5 *μ*s, and ΔP was recorded using a 12 bit ADC, conferring a resolution of 0.15 Pa. An example of the signal acquired with the NBAF system is showed in Figure 3. Considering the range of human walking speed, from 2 to 8 km/h, the NBAF system was designed to offer high sensitivity at this speed regime. Indeed, according to the theoretical relationship between air speed and ΔP (Equation 1), the system was able to cover a range of ±30 km/h within a full scale range of the pressure sensor of ±250 Pa.

The tri-axial accelerometer was used to measure body acceleration during movement. It consisted of a capacitive inertial sensor with a full scale of ±2 g (LIS3LV02DL, STMicroelectronics) with embedded a 12 bit ADC. The working principle of this sensor is based on the change in capacitance proportional to the forces applied to a mass which is displaced from its nominal position during motion. This causes an imbalance in a capacitive half-bridge, which can be measured by charge integration in response to a voltage pulse applied to the system.

Data for both pressure and acceleration sensors was acquired at a frequency of 20 Hz, saved in the memory card and downloaded via serial communication to personal computer at the end of the acquisition for post processing.

### 2.2. Protocol

Five adult male subjects voluntarily participated in the study. The subjects’ characteristics are presented in Table 1.

The test was composed by three parts. The aim of the first part was to determine the average step length of each participant during walking. The experiment took place indoors, in a straight and levelled corridor. The subjects were asked to walk twice at constant pace along a 60 meters path at a self-selected speed, during which the number of steps taken were counted by direct observation from the research staff. The step length (*l_step_*) was calculated as:

(2) $$l_{\mathrm{step}}\;=\;\frac{60m}{n_{\mathrm{step}}}$$

where *n_step_* was the number of steps taken to walk along the 60 meters path.

The second part of the experiment was conducted to measure NBAF and acceleration of the body during walking indoors. The participants were asked to walk twice (back and forth) on a 60 meter straight corridor at the fixed speed of 4, 5 and 6 km/h. This was done by instructing the participants to walk according to a step frequency imposed by an electronic metronome, which permitted to uniform the walking speed among the volunteers, assuming the individual step length as equal to the one measured during the first test. The step frequency (*f_step_*) was calculated with to the formula:

(3) $$f_{\mathrm{step}}\;=\;\frac{v}{l_{\mathrm{step}}}$$

where *l_step_* was the step length, and *v* the desired walking speed.

The third part of the test was carried out outdoors. The participants were asked to walk twice (back and forth) along a straight and flat sidewalk of 60 meters (path orientation: 74°). Also in this part of the test the walking speed was controlled by using a metronome to make the subjects walk at 4, 5, and 6 km/h. The aim of this outdoors trial was to test the ability of the NBAF system to predict walking speed in the field, where external circumstances, such as atmospheric conditions, can reduce the accuracy of the system.

During these tests, the body acceleration and the ΔP generated by NBAF were measured using the activity monitor. The device was placed around the waist close to the hip of the participants using an elastic belt. The air duct, which was mechanically fixed to the case of the activity monitor, was oriented along the direction of walking. In addition, the apparent walking speed was measured in each part of the test with a stopwatch. As a separate trial, measurements were repeated outdoors in the absence of movement over an epoch of 30 minutes to characterize the effect of wind on the sensor output (path orientation: 74°, atmospheric conditions: sunny, wind direction and speed: 160°, 2.1 m/s).

### 2.3. Data analysis and statistics

The acceleration signal was processed to calculate activity counts (AC) by summing up the absolute value of the acceleration recorded for each sensing axis in epochs of one minute. The AC measured during the entire duration of each walking task was averaged to obtain a mean AC value for every walking speed. Linear regression analysis was used to develop linear models for estimating walking speed using as independent elements AC.

**Figure 4.**
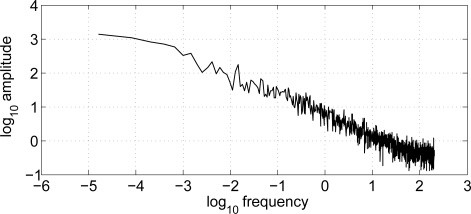
Power spectral density of the ΔP signal measured in absence of movement and caused by the air flowing inside the duct because of wind.

Theoretically, the relationship presented in Equation 1 could be used to determine walking speed and near-body air speed from measurements of ΔP. However, the real NBAF system differed from the theoretical model, because of the presence of noise sources that could not be easily eliminated. Indeed, atmospheric conditions, like the wind, represent an important source of noise to the ΔP signal measured during walking outdoors. It was observed that the power spectrum of the pressure sensor signal in outdoors condition decreased with increasing frequency, according to an inverse-exponential relationship with frequency (∼ 1/*f*^2/3^) (Figure 4). This behavior is typical of the noise occurring in many physical systems, including some meteorological data series. From the separate trial, it appeared that the power spectrum of the ΔP signal generated by the air flow produced by wind had components at frequency bands overlapping with the frequency components of the ΔP signal caused by ambulation. For this reason, a simple filtering technique was not able to separate the two contributions (wind and walking) in the ΔP signal measured outdoors.

The ΔP signal measured by the NBAF system was processed in two ways to assess walking speed. This two methods differed by the type of wind correction chosen for reducing the contribution of atmospheric conditions to the measured ΔP. The first method (Method-1) was based on the subtraction of the average ΔP value measured during the idle state, which preceded each walking task, from the ΔP signal recorded during walking. This method was based on the assumption that the wind was constant in magnitude and direction during the entire duration of the walking task. The squared root of the wind-corrected and averaged ΔP was then used to estimate walking speed by using linear regression analysis.

**Figure 5.**
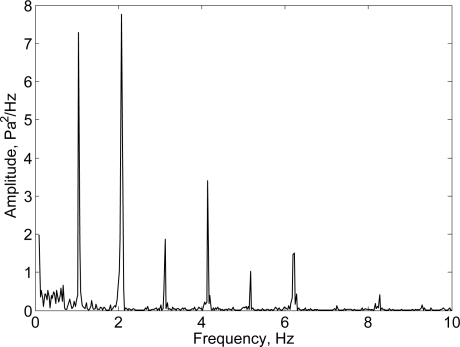
Power spectral density of the ΔP signal recorded during walking indoors at 2 Hz *f_step_*. From the figure it is possible to identify spectral peaks related to step and stride frequency.

The second method consisted in the separation of the contribution of wind and ambulation to the ΔP signal by spectral analysis. As showed in Figure 5 the ΔP signal presented clear peaks originated by the repetitiveness of certain phases of the stride cycle. These peaks were located at harmonic frequencies of the *f_step_*/2. Interestingly, the location of the peaks was not influenced by any external contribution. As observed, the wind altered, according to the direction and intensity, the magnitude of the harmonics due to stride cycle but not their position. The localization of the slowest peak (*f_peak_*) was the variable extracted from the ΔP signal power spectrum which was used to estimate walking speed with linear regression analysis.

Although the *f_step_* was imposed during the experimental protocol, the localization of *f_peak_* was automated by using an empirical algorithm, which was aimed at detecting the higher frequency peak within the range 0.7–1.38 Hz, whose boundaries have been set accordingly to the observations. In the performed test, this localized peak corresponded to the *f_step_*/2 peak of the ΔP power spectrum.

## 3. Results

**Figure 6.**
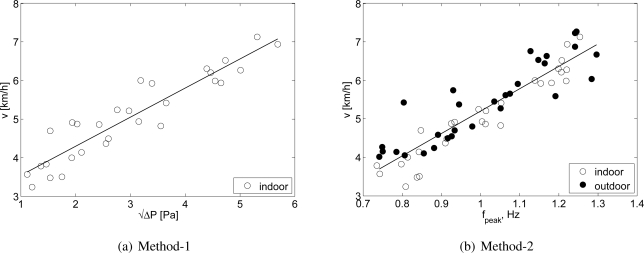
Relationship between measured walking speed and **(a)** $\sqrt{\Delta P}$ (Method-1), (**b**) *f_peak_* (Method-2).

The walking speed self-selected by the participants during the first part of the test was 4.8 ± 0.4 km/h and the measured step length was 0.72 ± 0.04 m. The step frequency during the second and third part of the test was set to 1.56 ± 0.09, 1.93 ± 0.11 and 2.34 ± 0.13 Hz, and the resulting walking speed was 4.0 ± 0.4, 5.2 ± 0.4 and 6.5 ± 0.5 km/h, respectively. In Table 2 is presented the results of the linear models based on AC to predict walking speed in the indoors, outdoors, and both conditions. In Table 3 are presented the prediction models of walking speed for indoors, outdoors, and both conditions based on the wind-corrected ΔP (Method-1) and the *f_peak_* (Method-2). The prediction model for outdoors using Method-1 was not significant (Figure 6(a)), because the wind correction technique was not successful. Method-2 provided a robust correction for wind effects as the prediction accuracy of walking speed was consistently high in all the developed models (SEE < 0.5 km/h, R^2^ > 80%) (Figure 6(b)).

**Figure 7.**
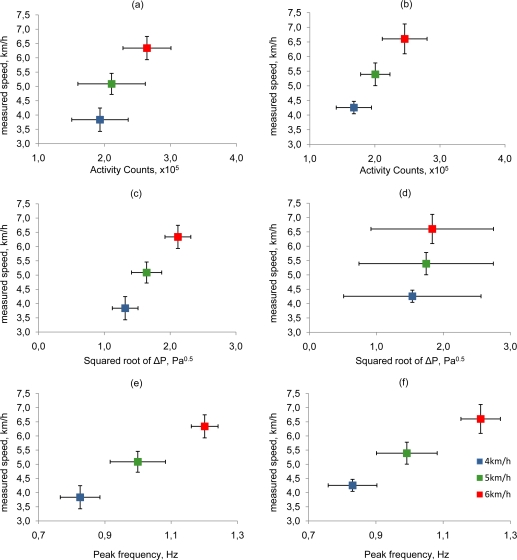
Representation of inter-individual variability in measured parameters indoors (a,c,e) and outdoors (b,d,f). Error bars indicate the standard deviation of the statistical distribution.

The between-subjects variability in AC was higher than the wind corrected ΔP (Method-1) in indoor conditions. The standard deviation of AC was 22%, 24%, and 14% of the average AC value for the three walking speed. On the other hand, the standard deviation of the wind-corrected ΔP was 15%, 14%, and 9% of the average value for each walking speed. The standard deviation of the *f_peak_* parameter (Method-2) was 7%, 8%, and 3% of the average value for each walking speed. This indicates that AC was more influenced by subjective differences in gait characteristics than the two measures of walking speed derived from the NBAF. However, this was not consistent in outdoors conditions. Indeed, during the third part of the test the standard deviation of the wind-corrected ΔP was much higher than indoors, because of the ineffective adjustment for the effect of the wind direction and magnitude in the measured signal. Outdoors, the standard deviation of AC was 16%, 11%, and 14% for the three walking speeds; the standard deviation of the wind-corrected ΔP (Method-1) was 51%, 41%, and 33% of the average value for the three walking speed, and the standard deviation of the *f_peak_* parameter (Method-2) was 9%, 9%, and 5% of the average value for each walking speed (Figure 7).

## 4. Discussion

This study reported that measurements of near-body air flow showed promising performance for accurately predicting walking speed. Depending on the method used to correct for the influence of wind to the ΔP measured with the NBAF system, different speed estimation accuracy was achieved. During the second part of the test, performed indoors, the ΔP signal was mainly generated by ambulatory movements, and both corrections Method-1 and Method-2 allowed the development of highly accurate prediction models. The ΔP signal recorded during walking outdoors was importantly influenced by wind and atmospheric conditions which were altering the flow of air inside the duct of the NBAF system. On this dataset, only correction Method-2 seemed to separate the ΔP signal resulting from ambulation from that caused by wind. On the other hand, activity counts showed poorer accuracy in predicting walking speed in both indoors and outdoors tests as compared to NBAF measurements. The reason was identified in the higher inter-individual variability in activity counts for a specific walking speed. Whereas the activity counts measured from the body acceleration were sensitive to differences in walking stride characteristics, the NBAF system was hypothesized to improve the prediction accuracy of walking speed because of a relatively higher independence of the output signal from subjects-specific variability in gait pattern. Ideally, a system able to objectively determine walking speed should be able to measure the horizontal displacement of the body over time, and therefore, be insensitive to the biomechanical effects of the stride cycle on different body segments. Indoors, the NBAF system showed to produce measurements that were highly reproducible between subjects as the coefficient of variation did not exceed 15%. However, outdoors measurements of the ΔP signal were significantly altered by external atmospheric conditions which reduced the prediction accuracy of walking speed and increased inter-individual variability.

Accelerometers have been previously used to estimate walking speed in laboratory and free-living conditions. The activity counts measured with a single accelerometer have been used to determine ambulatory speed during level walking [24,25] and during walking on slopes [26]. Schutz *et al.* [24] reported a large inter-individual variability in the relationship between accelerometer output and speed. Generic speed prediction models showed SEE bigger than 1 km/h, but individual calibration resulted in more accurate estimation of walking speed (SEE = 0.5 km/h). Barnett *et al.* [25] achieved slightly better estimation accuracy of speed using an accelerometer. A SEE below 0.6 km/h was achieved using the output of the MTI accelerometer (Actigraph, Pensacola, FL) to estimate walking speed. Yet, individual calibration decreased SEE to 0.2 km/h. Herren *et al.* [27] reported that an artificial neural network algorithm, after individual calibration, predicted walking speed with a SEE of 0.43 km/h. Speed prediction models based on temporal and spectral features of the acceleration signal offered highly accurate estimation of walking speed (SEE = 0.2 km/h) coupled with information on body characteristics [9]. To apply these prediction models in free-living conditions, the identification of activity types using physical activity recognition is required. A different approach to estimate walking speed using accelerometers data was based on detection of foot-ground contact events by identifying specific pattern of the trunk acceleration signal [20]. Information on stride duration was integrated with estimated step length to predict walking speed. However, treadmill tests revealed that subject-dependent correction factors were necessary to obtain accurate predictions of speed [19]. Applying individual correction factors to this model the SEE was between 0.11 and 0.25 km/h depending on the actual speed.

Global positioning systems have been proposed as tools to objectively determine displacement of the body during locomotion and consequently velocity. Schutz *et al.* [21] showed that a differential GPS estimated walking speed with SEE below 0.08 km/h, which suggests that this instrument is among the most accurate for estimating ambulatory speed. However, GPS are highly obtrusive and excessively power consuming for portable applications. In the last few years low-cost and low-power GPS have been developed in combination with accelerometers to estimate horizontal speed during locomotion. Tan *et al.* [23] showed that this system can produce estimation of running speed with a SEE of 0.54 km/h, independently from subjective variability in sensor output.

**Figure 8.**
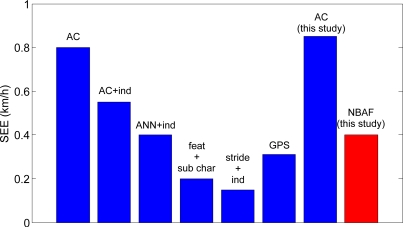
Estimation error of walking speed in the current and previous studies. AC, model based on activity counts [24,25]; AC + ind, model based on activity counts after individual calibration [24,25]; ANN + ind, model based on artificial neural network algorithm after individual calibration [27]; feat + sub char, model based on acceleration features and subject characteristics [9]; Stride + ind, model based on stride characteristics after individual calibration [19]; GPS, model based on GPS data [21,23]; AC, model based on activity counts presented in this study; NBAF, model based on near-body air flow.

In our study, the prediction models based on activity counts presented a SEE between 0.8 and 0.9 km/h in indoors and outdoors conditions. Thus, it was comparable to the accuracy achieved in many studies where prediction models were developed without individual calibration [24,26]. Quantifying near-body air flow by measuring the ΔP generated inside the air duct of the Pitot system allowed to significantly improve the estimation of walking speed as compared to AC. The SEE of the prediction models based on the wind-corrected ΔP was 0.4 km/h for indoors measurements. The *f_peak_* of the ΔP spectral components, related to stride frequency oscillations, could predict walking speed indoors and outdoors with a SEE between 0.3 and 0.5 km/h. Similar walking speed prediction accuracy could theoretically be achieved with any system able of determining step frequency. Although the *f_peak_* was able to accurately estimate speed, the presented model could show poorer accuracy when applied to different study populations, because of the inter-individual variability in stride length which determines ambulatory speed for a given stride frequency. Furthermore, the need of analysing the NBAF signal using FFT to determine *f_peak_* reduces the temporal resolution for estimating walking speed. Few strides are necessary before a sufficient number of samples are collected to determine *f_peak_* with sufficient spectral resolution. A summary of the walking speed estimation error as presented in the current and previous studies is depicted in Figure 8.

The limitation of this study was that a small study population was used to test the ability of the near-body air flow system in estimating walking speed. The study population should be characterized by broad range of age and height to validate whether subjects characteristics can negatively influence the accuracy of the models. In addition, daily life tests should be conducted to evaluate the accuracy of the system in predicting walking speed during short walks, and during walking in free-living conditions. Furthermore, a more robust wind correction method should be investigated. For example, advanced data processing techniques, such as wavelet analysis, or the detection of novel features in the ΔP signal might reduce the sensitivity of measurement to atmospheric conditions. A practical limitation of the developed activity monitor was that it required to be attached to the outside of the clothing for sensing the flowing air caused by locomotion. In addition, future studies are needed to investigate whether incorrect placement of the air duct with respect to the direction of movement can negatively influence sensor output. However, the measuring system allowed objective assessment of indoors ambulatory speed, which represents a positive feature of the proposed solution because accurate GPS systems cannot be used within buildings. Thus, an activity monitor equipped with a near-body air flow system might represent an optimal solution for accurately estimating indoors walking characteristics. Future studies are needed to investigate whether integrating the NBAF system with other sensors such as accelerometer or GPS can potentially improve the intrinsic performance for estimating walking speed in both indoor and outdoor conditions.

Monitoring and detecting trends in walking speed in daily life can improve prevention and diagnosis of the exacerbation of chronic diseases. Moreover, it can help improving the estimation accuracy of energy expenditure in free-living conditions [28]. Measurements of near-body air flow using a Pitot tube and a differential pressure sensor showed to decrease the estimation error of walking speed as compared to activity counts. This technology would certainly improve the accuracy of wearable sensors aimed at monitoring locomotion speed indoors. However, outdoors application of this technology should be further investigated to reduce the sensitivity of the system to atmospheric conditions.

## Figures and Tables

**Table 1.** Subjects’ physical characteristics (n = 5).

**Characteristic**	**Mean ± SD**
**Weight,***kg*	79.8 ± 16.3
**Height,***cm*	179.6 ± 7.3
**BMI,***kg/m*^2^	24.6 ± 3.7
**Age,***years*	27.0 ± 3.0

**Table 2.** Walking speed prediction models based on activity counts.

**Location**	**Indep.**	**Coeff.**	**p**	**R^2^**	**SEE**
Indoor	INT	2.04	< 0.05		
	AC	1.36×10^−5^	< 0.05		
Model				42%	0.8km/h
Outdoor	INT	2.38	< 0.05		
	AC	1.48×10^−5^	< 0.05		
Model				37%	0.8km/h
General	INT	2.48	< 0.05		
	AC	1.30×10^−5^	< 0.05		
Model				33%	0.9km/h

INT, intercept of the model; AC, activity counts; p, significance level; SEE, standard error of estimate.

**Table 3.** Walking speed prediction models based on near-body air flow measurements.

**Location**	**Method 1**					**Method 2**				
	**Indep.**	**Coeff.**	**p**	**R^2^**	**SEE**	**Indep**	**Coeff.**	**p**	**R^2^**	**SEE**
Indoor	INT	0.65	<0,05			INT	−1.28	<0.05		
	$\sqrt{\Delta P}$	2.62	<0.05			f_peak_	6.31	<0.05		
Model				86%	0.4km/h				91%	0.3km/h
Outdoor	INT					INT	0.00	*ns*		
	$\sqrt{\Delta P}$					f_peak_	5.35	<0.05		
Model				*not significant*					80%	0.5km/h
General	INT					INT	−0.63	<0.05		
	$\sqrt{\Delta P}$					f_peak_	5.83	<0.05		
Model				*not significant*					83%	0.4km/h

INT, intercept of the model; $\sqrt{\Delta P}$, squared root of the differential pressure; f_peak_, peak frequency of the power spectral density of the differential pressure; p, significance level; SEE, standard error of estimate.
